# Tumor Lysis Syndrome: An Endless Challenge in Onco-Nephrology

**DOI:** 10.3390/biomedicines10051012

**Published:** 2022-04-28

**Authors:** Gabriela Lupușoru, Ioana Ailincăi, Georgiana Frățilă, Oana Ungureanu, Andreea Andronesi, Mircea Lupușoru, Mihaela Banu, Ileana Văcăroiu, Constantin Dina, Ioanel Sinescu

**Affiliations:** 1 Department of Nephrology, Carol Davila University of Medicine and Pharmacy, 020021 Bucharest, Romania; gabriela.lupusoru@umfcd.ro (G.L.); ioana-georgiana.calin@drd.umfcd.ro (I.A.); valentina-georgiana.fratila@drd.umfcd.ro (G.F.); oana-catalina.ion@drd.umfcd.ro (O.U.); ileana.vacaroiu@umfcd.ro (I.V.); ioanel.sinescu@umfcd.ro (I.S.); 2 Department of Nephrology, Fundeni Clinical Institute, 022328 Bucharest, Romania; 3 Department of Physiology, Carol Davila University of Medicine and Pharmacy, 020021 Bucharest, Romania; 4 Department of Anatomy, Carol Davila University of Medicine and Pharmacy, 020021 Bucharest, Romania; mihaela.banu@umfcd.ro; 5 Department of Nephrology, Sf Ioan Clinical Emergency Hospital, 042122 Bucharest, Romania; 6 Department of Anatomy, Ovidius University, 900527 Constanța, Romania; constantin.dina@gmail.com; 7 Center for Uronephrology and Kidney Transplantation, Fundeni Clinical Institute, 022328 Bucharest, Romania

**Keywords:** cancer, chemotherapy, toxicity, tumor lysis syndrome

## Abstract

Tumor lysis syndrome (TLS) is a common cause of acute kidney injury in patients with malignancies, and it is a frequent condition for which the nephrologist is consulted in the case of the hospitalized oncological patient. Recognizing the patients at risk of developing TLS is essential, and so is the prophylactic treatment. The initiation of treatment for TLS is a medical emergency that must be addressed in a multidisciplinary team (oncologist, nephrologist, critical care physician) in order to reduce the risk of death and that of chronic renal impairment. TLS can occur spontaneously in the case of high tumor burden or may be caused by the initiation of highly efficient anti-tumor therapies, such as chemotherapy, radiation therapy, dexamethasone, monoclonal antibodies, CAR-T therapy, or hematopoietic stem cell transplantation. It is caused by lysis of tumor cells and the release of cellular components in the circulation, resulting in electrolytes and metabolic disturbances that can lead to organ dysfunction and even death. The aim of this paper is to review the scientific data on the updated definition of TLS, epidemiology, pathogenesis, and recognition of patients at risk of developing TLS, as well as to point out the recent advances in TLS treatment.

## 1. Introduction

Patients with malignancies are at increased risk of developing acute kidney injury (AKI) due to multiple causes: vomiting related to chemotherapy, nephrotoxicity of antineoplastic drugs, or direct kidney involvement caused by the underlying malignancy or urinary tract obstruction. AKI may be severe enough to eventually require renal replacement therapies, which will increase the morbidity and mortality of these patients [1].

Tumor lysis syndrome (TLS) is the result of a series of events leading to the rapid death of a high number of malignant cells. Lysis of these cells leads to the release of intracellular ions and metabolic byproducts into the bloodstream, resulting in hyperuricemia, hyperkalemia, hyperphosphatemia, and hypocalcemia. All these disturbances may cause serious complications such as AKI, cardiac arrhythmias, seizures, and even death.

TLS is an oncological emergency with high morbidity and mortality, especially if the diagnosis is delayed and treatment measures are not instituted promptly [2]. The most important aspect is to rapidly identify the patients at risk for TLS, in order to start the proper prophylactic and curative treatment. It commonly occurs in patients with high-grade hematological malignancies, such as acute leukemia and Burkitt’s lymphoma, but also in large and rapidly growing solid organ tumors, especially after starting chemotherapy [3,4]. It is a life-threatening condition, being responsible for increasing the in-hospital mortality of the cancer patient by up to 79% in cases of acute myeloid leukemia (AML) during induction therapy [5,6]. It may occur either spontaneously, or after antineoplastic therapy such as conventional chemotherapy, corticosteroids, molecular-targeted therapy, immunotherapy, and even after radiotherapy and chemoembolization [7,8,9,10,11,12,13,14,15,16,17,18].

## 2. Definition and Classification

Hande and Garrow classified TLS in 1993 in two categories: laboratory and clinical TLS [19]. They used some specific parameters of which variation are usually observed during the first four days after starting antineoplastic therapy. Their definition was not including patients with spontaneous TLS, and it was modified by Cairo and Bishop in 2004 by summing up the clinical and laboratory changes that appear within 3 to 7 days after the initiation of chemotherapy, thus including patients who already have TLS at presentation, as well as those who are developing it later on (Table 1) [20]. Additionally, it is necessary to exclude other causes of AKI.

Howard and colleagues made several amendments to the Cairo–Bishop definition in 2011:

- changes of the laboratory parameters must be simultaneous within 24 h because the patient may develop one abnormality, and later on another, unrelated to TLS (e.g., hypocalcemia associated with sepsis);
- symptomatic hypocalcemia has to be a criterion for clinical TLS, even when the decrease in calcium level is less than 25% of baseline;
- a 25% variation of a parameter is significant for the diagnosis only if it causes symptoms or if the value is not within the normal range [3].

Moreover, according to some authors [7], the definition of renal dysfunction has to be the same as the AKI definition, meaning an increase in serum creatinine by ≥0.3 mg/dL or an increase to ≥1.5 times baseline or oliguria (defined as a decrease in urine output below 0.5 mL/kg/h for 6–12 h) [21]. This will help to distinguish the patients with pre-existing kidney impairment from those who develop AKI secondary to TLS.

## 3. Pathogenesis

**Figure 1:**
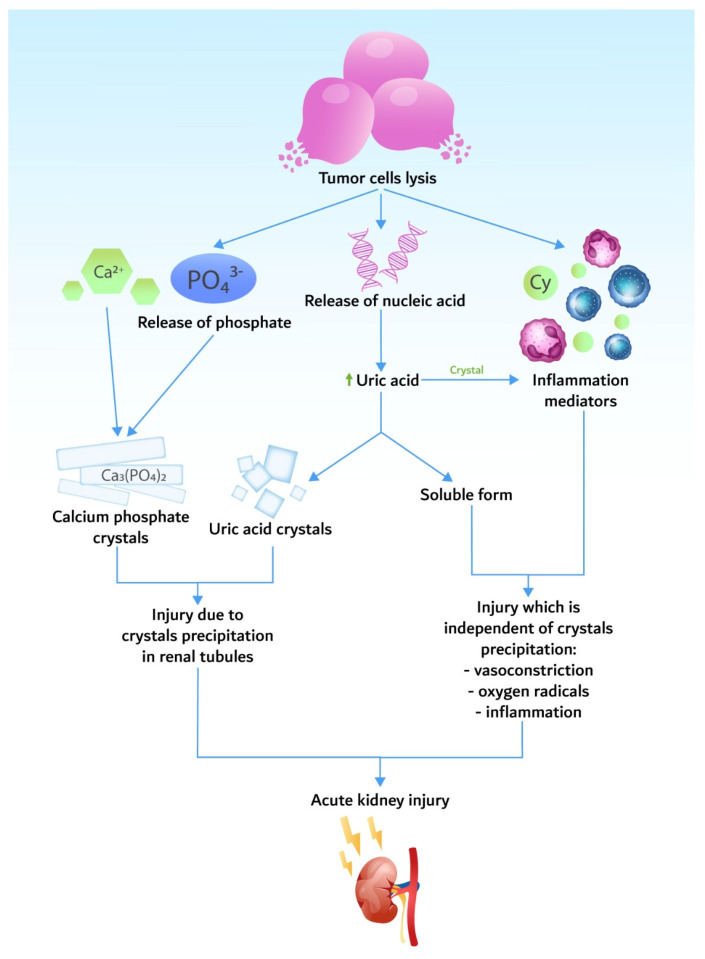
Pathogenesis of tumor lysis syndrome, resulting in acute kidney injury.

Pathogenic mechanisms are based on the release of potassium, phosphorus, and nucleic acids during tumor cell lysis in quantities higher than the body’s homeostatic mechanisms (Figure 1).

### 3.1. Hyperuricemia

**Figure 2:**
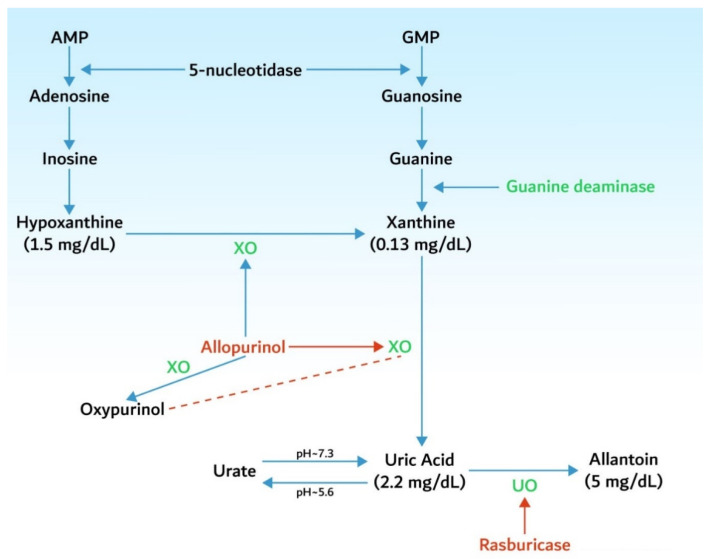
Uric acid metabolism and mechanisms of action of hypouricemic drugs. AMP—adenosine monophosphate; GMP—guanosine monophosphate; XO—xanthine oxydase; UO—urate oxydase.

Nucleic acids (adenosine monophosphate—AMP, guanosine monophosphate—GMP) are metabolized into adenine and guanine and then to hypoxanthine and xanthine, which is finally converted into uric acid under the influence of xanthine oxidase. In mammals, uric acid is further metabolized into allantoin (a molecule 5 to 10 times more soluble than uric acid) that is excreted by the kidneys. This catabolic pathway needs the presence of urate oxidase (OU), an enzyme lacking in humans and higher primates (Figure 2). Due to abnormal high levels, uric acid may precipitate into the renal tubules, especially in association with an acidic urine, contributing to renal dysfunction [22].

Tubular obstruction leads to progressive increase in proximal and distal tubule pressure and therefore to increased peritubular capillary pressure and vascular resistance. Thus, uric acid can cause kidney injury also through crystal-independent mechanisms, such as hemodynamic changes (renal vasoconstriction) [7,23] and impaired autoregulation caused by a low nitric oxide level with vasoconstriction [24]. Furthermore, high levels of uric acid cause smooth muscle cells to release cytokines that cause systemic inflammatory response syndrome: monocyte chemotactic protein 1 (MCP1), tumor necrosis factor (TNF-α), mitogen-activated protein kinases (MAPK), and nuclear factor kappa-light-chain-enhancer of activated B cells (NFkB) [25]. Proliferation of proximal tubular cells and endothelial cells is inhibited once renal injury is produced [26].

### 3.2. Hyperkalemia

Tumor cell lysis releases large amounts of potassium into the circulation, and the uptake capacity by muscle and liver is exceeded. It is even more pronounced in the setting of chronic kidney disease (CKD) or pre-existing AKI. It can lead to muscle fatigue, paralysis, arrhythmia, and death.

### 3.3. Hyperphosphatemia and Hypocalcemia

Cell lysis releases significant amounts of phosphate, leading to hyperphosphatemia. As in the case of hyperkalemia, hyperphosphatemia is even more severe in the setting of pre-existing kidney impairment. Malignant cells have a four times higher level of phosphate than normal cells [27]. It seems that hyperphosphatemia is less common in spontaneous TLS, as excessive phosphate is rapidly uptaken by the remaining highly metabolically active tumor cells [28,29,30,31].

High levels of serum phosphate can lead to precipitation of calcium phosphate in the renal tissue (nephrocalcinosis), especially in patients with an alkaline urine. Calcium phosphate can also precipitate in the conduction system of the heart, leading to conduction abnormalities and sometimes fatal arrhythmias. Hypouricemic therapy has evolved tremendously during the past years, so the major mechanism of AKI in TLS is represented by hyperphosphatemia and not by hyperuricemia. 

Another mechanism of phosphate toxicity is the binding of the calcium to the phosphate. Hypocalcemia may become symptomatic, causing neuromuscular excitability with tetany, seizures, arrhythmia, and death. Hypocalcemia may persist even after the resolution of hyperphosphatemia, possibly due to 1.25-vitamin D deficiency [32].

When a calcium x phosphate product is higher than 60 mg^2^/dL^2^, there is an increased risk of calcium phosphate precipitation in the renal tubules, leading to AKI. Calcium x phosphate product over 70 mg^2^/dL^2^ is an indication for dialysis.

## 4. Epidemiology

The incidence of TLS varies from sporadic cases up to high incidence (Table 2). However, even in tumors with low risk for TLS, the patient should be closely monitored as diseases such as multiple myeloma may develop TLS due to highly efficient modern anticancer therapy [33].

There is a great heterogeneity in reporting the incidence of TLS, mainly because most of the studies preceding the Cairo–Bishop criteria were retrospective and also because of the absence of uniform diagnostic criteria (some considering only clinical TLS as valid criteria).

A study including 788 adults and pediatric patients with acute leukemia or non-Hodgkin’s lymphoma demonstrated how incidence of TLS varied according to laboratory or clinical TLS—18.9% versus 5%, respectively. The incidences of laboratory and clinical TLS based on the tumor type were 21.4% and 5.2% in acute lymphoblastic leukemia (ALL), 14.7% and 3.4% in AML, and 19.6% and 6.1% in non-Hodgkin lymphoma, respectively [34]. In other studies, TLS incidence varied from 26% in ALL [35] to 32% in AML [36]. The highest risk of developing TLS is among patients with large and fast-growing tumors (e.g., Burkitt’s lymphoma B-cell ALL and AML with leukocytosis of over 100,000/mm^3^). The lowest incidence is among malignancies with a low multiplication rate, such as chronic leukemia and multiple myeloma. Large tumors (over 10 cm) and those with local invasion or metastases also have a high risk of TLS. Cases of catastrophic TLS in low-risk tumors are also described, for example, the reported cases of a patient receiving cetuximab for metastatic colon cancer [37], a patient with chronic leukemia who developed TLS during the first 24 h after starting imatinib [38], and a patient with renal cell carcinoma treated with sunitinib [39]. To the contrary, the TLS incidence may be significantly reduced in high-risk tumors (e.g., Burkitt’s lymphoma) due to aggressive prophylactic therapy with hydration and Rasburicase.

Tumor sensitivity to chemotherapy, tumor extension and metastases, and associated clinical conditions (CKD, hypovolemia, hypotension) also contribute to increased risk of TLS. 

## 5. Identification of Patients at Risk

TLS is associated with significant morbidity and mortality. Proper assessment of the patients with appropriate risk stratification is of major importance for a more efficient therapeutic approach. Several risk-stratification models for TLS have been developed [41,42], most of them taking into account different patients’ characteristics (including other comorbidities) and type of neoplasia (Table 3).

### 5.1. Tumor Risk Factors

Tumor-related risk factors include the type of neoplasia, sensitivity to chemotherapy, tumor volume, tumor growth rate (LDH level of more than two times the NV), and leukocytosis higher than 25,000/mm^3^ (Table 3).

Increased incidence of TLS is observed in tumors with high sensitivity to chemotherapy, such as endometrial cancer, hepatocellular carcinomas, or chronic leukemia (treated with biological therapy), cancers which are otherwise associated with low risk for this complication [3]. 

### 5.2. Risk Factors Related to Therapy

Intrathecal chemotherapy, interferon, steroids, radiation therapy, and bortezomib–cyclophosphamide–dexamethasone combination (in patients with multiple myeloma), as well as fludarabine, rituximab, or ibrutinib for chronic lymphocytic leukemia (CLL), are often associated with TLS [46,47].

Other substances and drugs (such as alcohol, caffeine, thiazide diuretics, acetylsalicylic acid, cisplatin, methyldopa, theophylline, pyrazinamide, diazoxide, ethambutol) that increase the level of serum uric acid can also increase the risk of TLS [48].

### 5.3. Tumor Lysis Syndrome Risk Stratification

The initial risk stratification model was based on the type of tumor and the level of leukocytosis [42]. Later on, other criteria were added, such as patient’s age, disease stage, histological aspects, anti-tumor therapy (cytotoxic agents and biological therapy), and kidney function [41,49].

Patients are broadly classified into three risk categories: high risk (>5% of patients develop TLS), intermediate risk (1–5% of patients develop TLS), and low risk (<1% of patients develop TLS).

A special situation is that of CLL, AML, and ALL, which are classified as high, low, or intermediate risk, respectively, according to the level of leukocytosis and LDH. Non-Hodgkin’s lymphomas are classified as high, intermediate, or low risk for TLS according to patient’s age, disease stage, and LDH level.

## 6. Tumor Lysis Syndrome Management

Management should focus on identifying patients at risk to develop TLS and on prompt prophylaxis. An adequate management has the purpose of reducing the risk of AKI and preventing the major electrolyte disorders. 

### 6.1. Prophylaxis

The key of prophylaxis is to maintain an adequate urine output and to decrease the blood levels of uric acid, potassium, and phosphate. The monitoring of biological values are recommended to be done with the following frequency:

- every 4 to 6 h after antitumor therapy initiation for patients at high risk;
- every 8 to 12 h for patients at intermediate risk;
- daily for patients at low risk.

In addition, it is recommended:

- to avoid the nephrotoxic drugs (NSAIDs, contrast agents);
- to stop the treatment with angiotensin-converting enzyme inhibitors and angiotensin receptor blockers.

#### 6.1.1. Volume Expansion

Volume expansion is accomplished by crystalloids solutions, which increase the urine output and thus the phosphate, potassium, and uric acid excretion. Apart from this effect, salt delivery to the distal tubules increases potassium secretion and lowers kalemia. Decreasing the urinary calcium x phosphate product also prevents the precipitation of the crystals. Therefore, it is recommended to administer 2500–3500 mL/m^2^/day to children (200 mL/kg/day for those whose weight is under 10 kg) and 3000 mL/day to adults by mouth or intravenously for 2 to 3 days before chemotherapy. The urine output that should be obtained is

- over 100 mL/m^2^/h or a diuresis of 2.5 L/day for adults;
- over 4 mL/kg/h for children.

#### 6.1.2. Diuretics 

Diuretics are not routinely recommended because they induce volume depletion, thus compromising the renal hemodynamics even more. Diuretics are used only in the case of symptomatic hypervolemia. The loop diuretics are preferred, because, in addition to increasing the urinary flow, they also increase the potassium secretion. Thiazides are contraindicated because they increase the blood levels of uric acid. 

#### 6.1.3. Urine Alkalinization

Urine alkalinization favors the conversion of the uric acid to urate and decreases tubular crystals precipitation (uric acid has a solubility of 0.15 mg/dL when pH is 5, whereas urate has a solubility of 2.2 mg/dL when pH is 7). However, alkalinization decreases calcium phosphate solubility and favors crystals precipitation in renal tubules and soft tissues. Moreover, alkalosis increases the amount of calcium that is bound to albumin and favors arrhythmia and tetany. Therefore, urine alkalinization is not recommended as it may even be dangerous. 

#### 6.1.4. Calcium Supplementation

Calcium is not routinely recommended, because it increases the precipitation of calcium in the soft tissues and it aggravates AKI. Calcium administration is recommended only in certain conditions: severe and symptomatic hypocalcemia (tetany, Chvostek sign, muscular fasciculation, bronchospasm, laryngospasm, seizures), changes of the electrocardiogram, and arrhythmia. In these cases, treatment is administered in order to alleviate the symptoms and not to normalize the calcemia. 

#### 6.1.5. Treating Hyperkalemia

Hyperkalemia must be promptly treated because it can induce life threatening arrhythmias. When potassium value increases with more than 25% compared to baseline value or when kalemia reaches 6 mmol/L, cardiac monitoring is recommended, altogether with the standard treatment: beta-adrenergic agonists (albuterol), glucose-insulin solution, short calcium gluconate infusion for myocardial protection, loop diuretics, and potassium binding resins in order to increase digestive loss. When kalemia exceeds 7 mmol/L, dialysis is recommended. Special attention is needed for the drugs that may increase the potassium serum values (Table 4).

#### 6.1.6. Allopurinol

Allopurinol is a purine analogue and is the isomer of hypoxanthine. It is metabolized by xanthine oxidase to oxypurinol (the active form of allopurinol), which is a competitive inhibitor of xanthine oxidase. Oxypurinol has a half time of 24 h and it is excreted by the kidneys, which makes the adjustments of the doses necessary in the case of kidney dysfunction. Oxypurinol decreases the production of uric acid from xanthine, but it has no effect on the uric acid that has been already synthetized [50]. This leads to a weak response to treatment in patients with TLS and severe hyperuricemia. Moreover, in this case, the serum concentration of hypoxanthine and xanthine increases after allopurinol and xanthine can precipitate into the renal tubules, leading to AKI. Therefore, it is recommended to use allopurinol as a prophylactic treatment and not in established TLS, where it can be used only if the patient is allergic to rasburicase or has glucose-6-phosphate dehydrogenase (G6PD) deficiency. 

Doses:

- for adults: 200–400 mg/m^2^/day, divided in 1 to 3 doses, to maximum 800 mg/day;
- for children: 300–450 mg/m^2^/day in 3 doses, to maximum 400 mg/day [41].

In chronic kidney disease, the dose is adjusted according to estimated glomerular filtration ratio (eGFR):

- 20–50 mL/min/1.73 m^2^: 200–300 mg/day;
- 10–20 mL/min/1.73 m^2^: 100–200 mg/day;
- <10 mL/min/1.73 m^2^: 100 mg/day or every 2 days.

Prophylactic therapy must begin at least 24 h prior to chemotherapy initiation and must be continued for at least 7 days. 

Allopurinol has multiple drug interactions; therefore, it is recommended to lower the doses for 6-mercaptopurine and azathioprine with 65–75% when these drugs are administered together with allopurinol. Moreover, other drugs for which doses should be adjusted in this situation are thiazides, cyclophosphamide, cyclosporine, ampicillin, and amoxicillin. 

Side effects are rare but may be life-threatening: from Steven–Johnson syndrome up to toxic epidermal necrolysis, acute toxic hepatitis, small vessel vasculitis, bone marrow aplasia, and DRESS syndrome (eosinophilia, rash, fever, lymphadenopathy, acute hepatitis, acute interstitial nephritis) [51]. Previous studies have shown that these side effects are idiosyncratic reactions [52,53]. 

#### 6.1.7. Febuxostat 

Febuxostat is a new inhibitor of xanthine oxidase. It does not induce the hypersensitivity reactions seen with allopurinol and it does not necessitate dose adjustment according to eGFR, so it is an alternative to allopurinol in certain groups of patients. The FLORENCE trial tested the efficiency of febuxostat compared to allopurinol in patients with blood malignancies and medium/high risk for developing TLS [54]. The study showed that febuxostat in a single daily dose of 120 mg/day was more efficient in preventing TLS. Another study found that febuxostat had the same efficiency as allopurinol in lowering serum and urinary uric acid levels in 45 children with ages between 5 and 15 years and hematological neoplasia, with medium and high risk of TLS [55]. In this study, febuxostat was administered in a single daily dose of 10 mg/day, a dose too small to demonstrate its better effect over allopurinol. A recent meta-analysis included six studies and was performed in order to evaluate the efficacy and safety of febuxostat compared to allopurinol as a prophylaxis for TLS; more than half of all the patients from this meta-analysis came from the FLORENCE trial. Both drugs showed a similar TLS incidence (OR 1.01, 95%CI: 0.56–1.81) and response rate (OR 1.01, 95%CI: 0.55–3.51) [56].

#### 6.1.8. Rasburicase 

Rasburicase is a recombinant variant of UO, derived from Aspergillus flavus. It was approved by the Food and Drug Administration (FDA) for pediatric patients with hematological malignancies undergoing chemotherapy in 2002 and for adults at risk for developing TLS in 2009 [57]. Rasburicase can be used for TLS prevention in high-risk patients and for the treatment of already-constituted TLS.

The efficacy and safety of rasburicase for the prevention and treatment of TLS in high-risk patients have been tested in a series of studies (Table 5) [58,59,60,61,62,63,64,65,66,67,68,69,70,71,72]. In one of them, it was tested in terms of its efficiency in treating hyperuricemia in patients with malignancies (1069 adults and children) and showed lower uric acid level in 99% of children and 100% of adults. Renal replacement therapies were necessary in only 2.8% of cases [73]. In a phase III study on patients at risk for TLS, Cortes et al. evaluated the uric acid lowering effect of rasburicase versus rasburicase followed by allopurinol administration, versus allopurinol. The study showed a better response for the patients treated exclusively with rasburicase. The uric acid was reduced in 87% of cases treated with rasburicase, versus 78% cases treated with rasburicase followed by allopurinol, versus 66% of cases treated with allopurinol [58]. 

Rasburicase converts uric acid in allantoin, carbon dioxide and hydrogen peroxide. Accumulation of hydrogen peroxide in patients with G6PD deficiency leads to methemoglobin accumulation leading to hemolytic anemia [74]. Prior to starting rasburicase administration, patients should be tested for G6PD deficiency. An initial dose of 0.05 mg/kg can be administered intravenously whenever testing is not possible. Regardless of this, these patients must be closely monitored for signs of hemolytic anemia. According to the guidelines for managing TLS in adult and pediatric patients with hematological malignancies elaborated by the British Committee of Standards in Haematology, it is recommended to administer rasburicase as a prophylaxis to high-risk patients in a single dose of 3 mg to adults and 0.2 mg/kg to children. Rasburicase administration can be repeated daily for 5–7 days when necessary (lack of response or increase in the uric acid level) [48].

Rasburicase is very well tolerated with only few side effects, has less drug interactions compared to allopurinol, and has a rapid effect (it lowers uric acid level in 4 h). Rarely, it can induce rash, fever, headache, nausea, vomiting, or hepatic cytolysis and it is contraindicated in pregnancy, lactating women, or patients with G6PD deficiency. Rasburicase continues to be active ex vivo, thus leading to false decreased values of uric acid if the blood sample is not immediately delivered to the laboratory in ice recipients in order to be processed in no more than 4 h after blood collection.

#### 6.1.9. Treating Hyperphosphatemia

The main therapeutic measure is the increase in phosphaturia by volume expansion with isotonic solutions. The management should also focus on dietary phosphorus restrictions and the administration of oral phosphate chelating agents (e.g., sevelamer). Dialysis is indicated when the value of the calcium x phosphate product exceeds 70 mg^2/^dL^2^, despite the therapeutic and prophylactic measures.

#### 6.1.10. Future Perspectives

##### Prevention through Chemotherapy Modulation 

The risk of TLS can be diminished through choosing less aggressive chemotherapy regimens in order to slowly reduce the tumor burden, allowing enough time for the compensatory kidney mechanisms to remove the products derived from tumor lysis. For example, the Berlin–Frankfurt–Muenster group treated a child with ALL by initially administering prednisone for one week before initiating induction chemotherapy [75]. For patients with Burkitt’s lymphoma, there are regimens that include a first week of treatment with low doses of vincristine, prednisone, and cyclophosphamide, followed by full doses.

In the future, TLS prophylaxis may be improved by means of interfering with malignant cells multiplication and metastasis. For example, it is possible to decrease the invasive behavior by targeting the expression of desmosome junction proteins through drugs that increase the expression of these desmosomes [76]. Desmosomes are intercellular junctions whose main function is strong adhesion; +9most of the mechanisms that regulate their assembly and stability in migratory epithelial cells are still unknown [77]. A series of studies have reported a correlation between reduced desmosome expression and the invasiveness of tumors, such as transitional cell carcinoma of the bladder and non-small cell lung cancer, conditions that may be associated with TLS [78,79,80], and thus it is too premature to close the chapter focusing on the prophylaxis of TLS.

##### Other Options for Enzyme Replacement Therapy 

Due to the limited efficiency of xanthine oxidase inhibitors and side effects of intravenous rasburicase, during the past years, studies were aimed at developing oral engineered uricase. Although the kidney is the major site of elimination for uric acid, the gut also plays an important role in urate metabolism. Oral recombinant urate oxidase (ALLN-346), which targets intestinal degradation of urate, was studied in mice deficient in uricase with severe hyperuricemia and crystalline obstructive nephropathy [81]. ALLN-346 determined a significant reduction of hyperuricemia by 44% and the normalization of uricosuria.

Pegloticase is a third-line treatment that was authorized by the European Medicines Agency in 2016 for the treatment of severe, refractory gout. It is given as an intravenous infusion every two weeks. Pegloticase is a recombinant porcine uricase that is pegylated to increase its elimination half-life and to decrease the immunogenicity. Like rasburicase, it is contraindicated in patients with G6PD deficiency. NCT04745910 is a phase-IV clinical trial that will soon start patients’ recruitment. Its aim is to assess the efficiency of pegloticase compared to rasburicase in reducing uric acid levels in patients with hyperuricemia caused by TLS [82]. 

### 6.2. Treatment

Once established, TLS necessitates a multidisciplinary approach and a careful monitoring of some key elements: regularly checking the patient because his general status may change from one hour to another, and monitoring diuresis, laboratory tests, and possible complications. 

It is recommended to maintain a urine output of at least 100 mL/m^2^/h for adults and 4 mL/kg/h for children. Urine alkalinization is not recommended (level 1C recommendation) [48]. 

It is recommended to administer rasburicase and not xanthine oxidase inhibitors, since they do not have any effect on the uric acid that has already been produced. The only indications for xanthine oxidase inhibitors are known rasburicase allergy and G6PD deficiency [48]. As a therapy, the recommended dose of rasburicase is 0.2 mg/kg/day, and the treatment duration must be established according to clinical response, but no more than 3 to 7 days. In contrast to the prophylactic treatment, when administration of low doses of rasburicase is supported by multiple studies, there are not enough data to support a fixed, low dose of rasburicase compared to weight adapted doses when dealing with an already constituted TLS. Some studies still recommend a fixed, single dose of 6 mg, which was found to be as effective as the weight-adapted dose [74]. Therefore, for financial reasons, this alternative treatment may be reasonable and suitable for daily administration. 

#### Renal Replacement Therapy

The necessity for renal replacement therapy (RRT) decreased dramatically in the era of hypouricemic drugs, especially in the countries where rasburicase is available for the prevention and treatment of TLS. RRT is indicated when kidney dysfunction is aggravating despite therapeutic measures, when the patient develops hypervolemia, or when electrolyte disturbances are refractory to medical treatment. 

The options for RRT are:

- daily hemodialysis;
- continuous veno-venous hemofiltration;
- combination of intermittent hemodialysis and continuous hemofiltration/hemodiafiltration for an efficient clearance of phosphate, which is time dependent. These techniques use dialysis membranes with large pores, which allow for rapid clearance of molecules that otherwise are not efficiently removed by conventional hemodialysis.

Peritoneal dialysis is not adequate, because it offers a less efficient clearance of uric acid and phosphate. Moreover, patients may associate abdominal complications related to neoplasia (peritoneal carcinomatosis, compartment syndrome), which are contraindications for this procedure. 

When necessary, RRT should be performed until urine output and electrolyte values return to normal.

## 7. Conclusions

TLS is a constellation of metabolic disorders that occur as a result of malignant cells lysis, related either to the treatment or to the increased rate of cells proliferation. TLS is an onco-nephrology emergency, with AKI being one of the most important predictors of short- and long-term mortality in these patients. According to TLS risk stratification, an early, aggressive, and multidisciplinary approach is mandatory in order to limit the occurrence of this condition and consequently of AKI. Nowadays, new advances in chemotherapy pose the risk of TLS developing even in patients with malignancies that were previously classified as having a low risk for this complication. 

## Figures and Tables

**Table 1:** Cairo–Bishop criteria for defining tumor lysis syndrome (modified after [20]).

Cairo–Bishop Definition of Tumor Lysis Syndrome			
Laboratory TLS = modification of at least 2 parameters within 24 h	- Uric acid ≥ 8 mg/dL - Potassium ≥ 6 mg/dL - Phosphate ≥ 4.5 mg/dL	Or 25% increase	within 3 to 7 days after chemotherapy initiation
	- Calcium ≤ 7 mg/dL	Or 25% decrease	
Clinical TLS = laboratory TLS + 1 organ dysfunction or death	- Renal dysfunction (creatinine > 1.5 X normal values) - Cardiac involvement (arrhythmias) - Neurological involvement (seizures, tetany) - Death		

TLS—tumor lysis syndrome.

**Table 2:** Solid tumors associated with tumor lysis syndrome [40].

Germ cell tumors
Neuro- and medulla blastomas
Small cell carcinoma and other lung tumors
Breast, ovarian, and vulvar neoplasms
Hepatoblastoma and hepatocellular carcinoma
Colorectal and gastric carcinoma
Melanoma
Sarcoma

**Table 3:** Risk factors for tumor lysis syndrome [6,34,43,44,45].

Tumor Risk Factors	Patient-Related Risk Factors
Type of tumor	Male gender
Tumor volume (tumors > 10 cm)	Age > 65 years
Metastatic disease	Pretreatment serum creatinine > 1.4 mg/dL
Tumor growth rate (LDH > 2 times NV)	Renal obstruction
Level of leukocytosis (>25,000/mm^3^)	Pretreatment serum uric acid > 7.5 mg/dL
Sensitivity to chemotherapy (germ cell tumors, small cell lung cancer, etc.)	Associated conditions (hypotension, hypovolemia, nephrotoxic drugs, CKD)

LDH—lactate dehydrogenase; CKD—chronic kidney disease; NV—normal value.

**Table 4:** Drugs that increase the serum potassium level.

**Drugs That Increase the Release of the Potassium from the Cells**
Beta-adrenergic blockers
Digoxin
Verapamil
Mannitol
**Drugs that interfere with the renin–angiotensin–aldosterone system**
Angiotensin-converting enzyme inhibitors and angiotensin receptor blockers
NSAIDs
Calcineurin inhibitors
Heparin
Potassium sparing diuretics
Trimethoprim
**Drugs that contain potassium**
G penicillin
Frozen blood products

NSAIDs—non-steroidal anti-inflammatory drugs.

**Table 5:** Studies evaluating the efficacy of rasburicase in the prevention/treatment of tumor lysis syndrome.

Author (Year)	Modality	Sample Size	Treatment	Primary Outcome(s)	Results and Key Observations
Cortes (2010) [58]	Multicenter, randomized, comparative phase III study	275 (adults)	Three treatment arms: - RSB (n = 92) - RSB and ALLP (n = 92) - ALLP (n = 91)	Percentage of patients achieving PUA ≤ 7.5 mg/dL during days 3–7 of treatment	PUA response rate: −87% with RSB, 78% with RSB and ALLP, 66% with ALLP. RSB was significantly better than ALLP in controlling PUA in terms of rapidity and efficacy.
Gopakumar (2017) [59]	Retrospective study	18 (children)	RSB—low, single dose (0.085 mg/Kg)	Decrease in PUA up to normal levels	PUA decrease: - 31.18% at 4 h - 64.8% at 24 h - 74.5% at 48 h RSB at a lower, single dose efficiently decreases PUA up to a normal level.
Kikuchi (2009) [60]	Multicenter, randomized, parallel-group study	30 (children)	RSB: - 0.15 mg/Kg/day for 5 days (n = 15) - 0.2 mg/Kg/day for 5 days (n = 15)	PUA ≤ 6.5 mg/dL (pts <13 years) or ≤ 7.5 mg/dL (pts >13 years) by 48 h after first administration, lasting 24 h after the final administration	Response rates: - 93.3% with RSB 0.15 mg/Kg/day - 100% with RSB 0.2 mg/Kg/day UA levels decrease within 4 h of starting RSB administration in both groups.
Vadhan-Raj (2012) [61]	Randomized, comparative, controlled, open-label trial	82 (adults)	RSB—two treatment arms: - 0.15 mg/Kg, single dose (n = 40); more doses (maximum 5) administered only if PUA > 7.5 mg/dL - 0.15 mg/Kg daily for 5 days (n = 40)		PUA response: - 79 pts (99%) within 4 h of starting RSB administration - 39 pts (98%) in daily-dose arm - 34 pts (85%) in single-dose arm Five pts within the single-dose arm required a second dose of RSB.
Tatay (2010) [62]	Randomized, comparative study	32 (children)	- ALLP 10 mg/Kg/day every 8 h (n = 16) - RSB 0.2 mg/Kg/day once daily (n = 16)	Testing efficacity of RSB versus ALLP in the treatment of hyperuricemia in TLS	- 4 h after the first dose, pts treated with RSB achieved a greater reduction of PUA levels compared to ALLP. - 56% of pts in the ALLP group needed dialysis and none from the RSB group.
Wang (2006) [63]	Multicenter, nonrandomized, open-label clinical trial	45 (18 children, 27 adults)	RSB 0.2 mg/kg/day for 2–6 day	Decrease in PUA up to normal level and preventing TLS	The median PUA levels decreased to 0.5 mg/dL in all pts, and none of the pts required dialysis.
Galardy (2013) [64]	Multicenter, randomized, open-trial	76 (children)	RSB 0.2 mg/kg/day, minimum 1 dose prior to cytoreduction therapy and every 24 h if needed for up to maximum 5 doses	Decrease in PUA to a normal level and preventing LTLS and CTLS	Following RSB there was 9% incidence of LTLS and 5% incidence of CTLS. There was a significant improvement in estimated GFR following RSB. Only 1.3% pts required dialysis.
Goldman (2001) [65]	Multicenter, randomized, comparative trial	52 (children)	- RSB (n = 27) - ALLP (n = 25)	RSB versus ALLP in decreasing PUA levels during the first 5 days of chemotherapy and the reduction of PUA at 4 h after the first dose of treatment	- Pts receiving RSB experienced a 2.6-fold less PUA during the first 96 h of therapy. - There was an 86% reduction in PUA levels after 4 h of the first dose of RSB compared to only 12% for ALLP.
Digumarti (2014) [66]	Multicenter, open-label, phase-III study	88 (adults)	RSB 0.2 mg/kg/day for 4 days	- Percentage of reduction in PUA at 4 h after RSB - PUA AUC (0–96 h) - Incidence of adverse events	- There was a 75.3 ± 28.5% of reduction in PUA at 4 h as compared to baseline. - The PUA AUC (0–96 h) was 259.9 ± 215.5 mg/dL/h. - 29 pts had mild-to-moderate adverse events.
Bosly (2003) [67]	Multicenter, randomized study	278 (166 children, 112 adults)	RSB 0.2 mg/kg/day for 1–7 days	Decrease in PUA to ≤6.5 mg/dL (pts < 13 years) or ≤7.5 mg/dL (pts > 13 years)	- There was a significant decrease in mean PUA level among both adult and pediatric pts, regardless of whether they were hyperuricemic at presentation or not. - After RSB treatment, all pts had PUA levels within the normal range; the response rate was 100%.
Vachhani (2021) [68]	Prospective, randomized study	24 (adults)	RSB—two treatment arms: - 1.5 mg—day 1 (Arm A) - 3 mg day 1 (Arm B) ALLP 300 mg on days 1–6 to all pts	The efficacity of two lower, single doses of RSB in decreasing UA levels	- 83% pts in both arms achieved PUA < 7.5 mg/dL by 24 h after therapy. - 21% pts required additional doses of RSB. - 23/24 of pts achieved UA goals after 1–2 doses of RSB.
Rényi (2007) [69]	Multicenter, prospective, open-label, phase-IV study	- 36 (children) - 14 pts (historic cohort)	- RSB 0.2 mg/kg for 5 days (n = 36) - ALLP 300 mg/m^2^ daily dose (historic cohort of 14 pts)	The efficacity of RSB to decrease UA level and prevent AKI in pts at risk for TLS	- RSB decreased the PUA level by 4 h after first dose of treatment from 343 micromol/L to 58 micromol/L. - ALLP significantly decreased PUA level by 61 h after treatment. - In the RSB group, 1 patient required dialysis compared to 3 pts that experienced AKI (1 requiring dialysis) in the ALLP group.
Coiffier (2003) [70]	Prospective, randomized study	100 (adults)	RSB 0.2 mg/kg/day for 3–7 days	Testing efficacity and safety of RSB in prevention and treatment of hyperuricaemia during induction chemotherapy of non-Hodgkin’s lymphoma	- RSB treatment controlled the PUA level within 4 h after first dose. - No patient experienced increased creatinine levels or required dialysis.
Shin (2006) [71]	Prospective, open-label, phase-IV study	37 (children)	RSB 0.2 mg/kg/day, once daily for 3–5 days	PUA ≤ 7 mg/dL and safety of RSB	- 36/37 (97.3%) pts reached PUA endpoint. - Drug-related toxicities were mild and reversible.
Malaguarnera (2009) [72]	Pilot randomized clinical trial (comparison of RSB and placebo)	38 (elderly)	- RSB 4.5 mg in 100-cc physiological saline or - Placebo (physiological saline)	Reducing PUA and improving renal function	- In the RSB group, mean PUA had decreased 93% at day 1, 73% at day 7, and remained within a normal value after 1 month of therapy. - 2 months after RSB, there was a significant reduction of urate and creatinine and an increase in creatinine clearance and urate clearance.

RSB—rasburicase; ALLP—allopurinol; n—number of patients; pts—patients; PUA—plasma uric acid; TLS—tumor lysis syndrome; LTLS—laboratory tumor lysis syndrome; CTLS—clinical tumor lysis syndrome; AUC—area under the curve; GFR—glomerular filtration ratio; AKI—acute kidney injury.

## Data Availability

Not applicable.
